# Clinical outcomes of iatrogenic upper gastrointestinal endoscopic perforation: a 10-year study

**DOI:** 10.1186/s12876-019-1139-1

**Published:** 2019-12-16

**Authors:** Dae Hwan Kang, Dae Gon Ryu, Cheol Woong Choi, Hyung Wook Kim, Su Bum Park, Su Jin Kim, Hyeong Seok Nam

**Affiliations:** Department of Internal Medicine, Medical Research Institute, Pusan National University School of Medicine and Research Institute for Convergence of Biomedical Science and Technology, Pusan National University Yangsan Hospital, Beomeo-ri Mulgeum-eup, Yangsan-si, Gyeongsangnam-do 50612 South Korea

**Keywords:** Perforation, Endoscopy, Resection, Iatrogenic, Mortality

## Abstract

**Background:**

Upper gastrointestinal endoscopic examination is a relatively safe procedure; however, all endoscopic procedures are invasive and are associated with a risk of iatrogenic perforation. To evaluate clinical outcomes of iatrogenic upper gastrointestinal endoscopic perforation. Factors associated with surgical management or mortality were analyzed.

**Methods:**

Between November 2008 and November 2018, the medical records of 149,792 upper gastrointestinal endoscopic procedures were evaluated. The mechanisms of perforations were categorized as electrocoagulation-induced or blunt trauma-induced injuries. The incidence and clinical outcomes of iatrogenic perforations based on the types of procedures performed were evaluated.

**Results:**

Iatrogenic endoscopic perforations occurred in 28 cases (0.019%). Iatrogenic perforation-related mortality occurred in 3 patients. The iatrogenic perforation rate based on the types of procedures performed was as follows: diagnostic endoscopy = 0.002%, duodenal endoscopic mucosal resection = 0.9%, esophageal endoscopic submucosal dissection = 10.7%, gastric endoscopic submucosal dissection = 0.2%, endoscopic self-expandable metal stent insertion for malignant esophageal obstruction = 0.1%, duodenoscope-induced injury = 0.02%, endoscopic sphincterotomy = 0.08%, and ampullectomy = 6.8%. All electrocoagulation-induced perforations (*n* = 21) were managed successfully (15 cases of endoscopic closure, 5 cases treated conservatively, and 1 case treated surgically). Three patients died among those with blunt trauma-induced perforations (*n* = 7). The factors associated with surgical management or mortality were old age, poor performance status (Eastern Cooperative Oncology Group score ≥ 1), advanced malignancy, and blunt trauma.

**Conclusions:**

Most cases of electrocoagulation-induced iatrogenic perforations can be treated using endoscopic clips. If endoscopic closure fails for blunt trauma-induced perforations, prompt surgical management is mandatory.

## Background

Upper gastrointestinal endoscopic examination is considered a relatively safe procedure; however, iatrogenic endoscopic perforations may necessitate emergency operations and may rarely be fatal. With the widespread popularity of diagnostic endoscopic examination, several therapeutic endoscopic modalities such as endoscopic mucosal resection (EMR), endoscopic submucosal dissection (ESD), endoscopic balloon dilation for benign strictures, endoscopic self-expandable metal stent (SEMS) insertion for malignant strictures, and endoscopic retrograde cholangiopancreatography (ERCP) related procedures are being commonly performed in clinical practice.

Early diagnosis and prompt management of iatrogenic endoscopic perforations reduce the morbidity and mortality rates. Surgical repair and drainage constitute the conventional treatment for gastrointestinal tract perforations. However, in recent years, various endoscopic instruments and techniques are used for closure of gastrointestinal wall defects using endoscopic clips, over-the-scope clips, and stenting [1]. Although the reported incidence of iatrogenic endoscopic perforations during diagnostic endoscopy is 0.0009–0.01% [2], the risk of perforation is increasing owing to procedural difficulties associated with endoscopy, anatomical sites of perforations, and endoscopists’ experience [3, 4].

No evidence-based guidelines are established for the management of iatrogenic endoscopic perforations because the choice of therapeutic modality used depends upon its availability in the hospital, physicians’ experience, anatomical sites of lesions, and the patients’ comorbidities. The timing of diagnosis of acute iatrogenic perforations is important because leakage of contaminated food or fluid into the thoracic or abdominal cavity may induce fatal infection. Compared with postoperative leakage or fistula formation, tissue around the site of an acute iatrogenic perforation is healthy, is easy to approximate using endoscopic clips, and heals readily. However, delayed diagnosis of a perforation leads to progressive inflammation around the site and consequent difficulty with endoscopic approximation. Factors that determine the optimal management of a perforation are the mechanism of iatrogenic endoscopic perforation (electrocoagulation- or blunt trauma-induced injury), patients’ performance status, anatomical sites involved, and the surgeon’s experience. Knowledge of factors associated with surgical management and mortality are important to decide the optimal treatment modalities to manage iatrogenic endoscopic perforations.

This 10-year study reviewed the clinical data and outcomes in patients with iatrogenic endoscopic perforation. The factors associated with surgical management or mortality were also evaluated.

## Methods

**Fig. 1:**
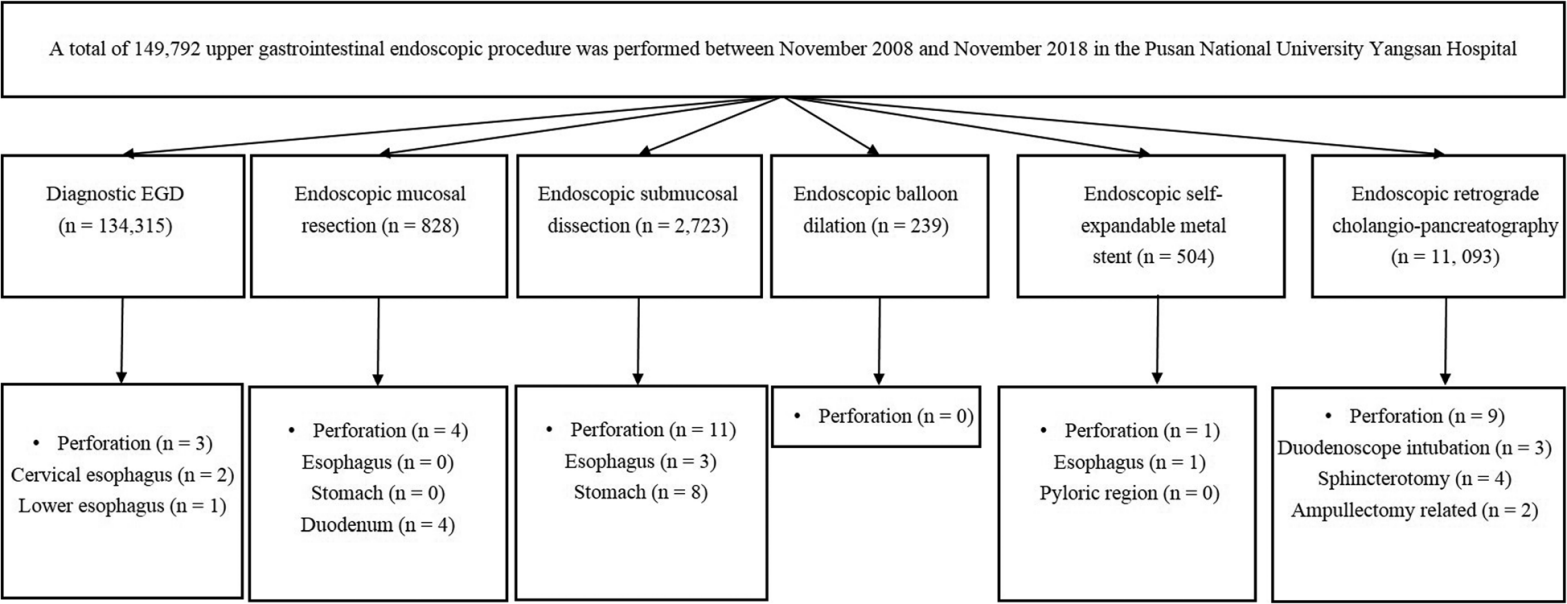
Flow chart showing the study design

The medical records of patients who underwent diagnostic or therapeutic endoscopic examination at the Pusan National University Yangsan Hospital, Republic of Korea between November 2008 and November 2018 were retrospectively reviewed. During this study period, the 149,792 upper gastrointestinal endoscopic procedures performed included the following: diagnostic endoscopy (*n* = 134,315), EMR (*n* = 828), ESD (*n* = 2723), endoscopic balloon dilation (*n* = 329), endoscopic SEMS insertion (*n* = 504) and ERCP-related procedures (*n* = 11,093). We observed 28 iatrogenic endoscopic perforations during this period (Fig. 1). Written informed consent was obtained from all patients prior to the endoscopic procedures. The present study was approved by the Ethics Committee of the hospital where this study was performed.

Data regarding patients’ characteristics, types of endoscopic procedures performed, and indications for the procedure were evaluated using retrospective chart review. Most endoscopic examinations and procedures were performed under conscious sedation using intravenously administered midazolam (0.05 mg/kg) and meperidine (50 mg). Data regarding the iatrogenic endoscopic perforation such as its location, the mechanism of injury, time until diagnosis, and management of the perforation were reviewed based on chart review, endoscopic images, and radiological examination. Daily living abilities of the patients were evaluated using the Eastern Cooperative Oncology Group (ECOG) performance status tool [5].

Most iatrogenic endoscopic perforations were diagnosed when a perforation was observed during the endoscopic procedure. The presence of free intra-abdominal air or subcutaneous emphysema detected by radiological examination indicated delayed presentation of a perforation. Endoscopic closure using clips was initially attempted following the diagnosis of an iatrogenic endoscopic perforation. If a microperforation without evidence of infection was identified, the patient was administered intravenous antibiotics and a nil per os status was maintained until resolution of infection and/or pain. Surgical management was recommended if endoscopic closure of the perforation was impossible or incomplete. Intravenous antibiotics were administered with total parenteral nutrition if the patient’s performance status was a contraindication for surgery or if a patient refused surgical repair. The mechanisms of iatrogenic endoscopic perforations were classified as blunt trauma-induced perforations (endoscopic tip or shaft- or SEMS-related injury) or electrocoagulation-induced perforations (endoscopic electrosurgical knife-, snare- or coagulation-related injury).

### Statistical analyses

Baseline characteristics of patients and the length of hospital stay are expressed as means (standard deviation) or medians (range). Chi-squared and t-tests were also used for analysis. A *p* value < 0.05 was considered statistically significant. All statistical analyses were performed using the SPSS statistical software, version 21.0 (IBM SPSS).

## Results

A total of 28 iatrogenic endoscopic perforations occurred during the study period (0.019%, 28/149,792). Esophageal ESD (10.7%) and ampullectomy (6.8%) were the leading causes of iatrogenic endoscopic perforations based on the types of endoscopic procedures performed. We observed that 0.002% of iatrogenic perforations occurred during diagnostic endoscopy. The incidence of iatrogenic endoscopic perforations based on the types of procedures performed is shown in Table 1. The baseline characteristics of patients presenting with iatrogenic perforations are shown in Table 2. The patients’ mean age was 65.5 ± 17.4 years, and the study group comprised 53.6% (15/28) men. The mean hospital stay was 10.0 ± 6 days. The sites of perforation were as follows: esophagus (*n* = 8), stomach (n = 8), and duodenum including ampulla (*n* = 12) (Table 2). Advanced malignancy was detected in 17.8% of patients. The ECOG performance status was 0 (85.7%) in most cases.

**Table 1:** Incidence of iatrogenic endoscopic perforations based on the types of procedures performed

	Total number of procedures, (n)	Perforation, n (%)
Diagnostic endoscopy	134,315	3 (0.002)
Endoscopic mucosal resection	828	4 (0.483)
Esophagus	19	0 (0)
Stomach	396	0 (0)
Duodenum	413	4 (0.968)
Endoscopic submucosal dissection	2723	11 (0.403)
Esophagus	28	3 (10.714)
Stomach	2695	8 (0.296)
Endoscopic balloon dilation	239	
Esophagus	186	0 (0)
Pyloric region	53	0 (0)
Self-expandable metal stent insertion	504	1 (0.198)
Esophagus	177	1 (0.564)
Pyloric region	327	0 (0)
ERCP-related procedures	11,093	9 (0.081)
Duodenoscopic intubation	11,093	3 (0.027)
Sphincterotomy	4580	4 (0.087)
Ampullectomy	29	2 (6.896)
Biliary stenting	731	0 (0)
Total	149,792	28 (0.019)

*ERCP* Endoscopic retrograde cholangiopancreatography

**Table 2:** Characteristics of patients with iatrogenic endoscopic perforations

Characteristics	Total (*n* = 28)
Age, years (mean ± SD)	65.5 (17.4)
Male sex, n (%)	15 (53.6)
Mean hospital stay, days (mean ± SD)	10.0 (6.0)
ECOG performance status, n (%)	
0	24 (85.7)
1	1 (3.6)
2	3 (10.7)
Advanced malignancy, n (%)	5 (17.8)
Pancreatic cancer	1 (3.6)
Cholangiocarcinoma	1 (3.6)
Duodenal adenocarcinoma	1 (3.6)
Esophageal cancer	1 (3.6)
Gastric cancer	1 (3.6)
Site of perforation	
Esophagus	
Cervical esophagus	2 (7.1)
Thoracic esophagus	1 (3.6)
Lower esophagus	5 (17.8)
Stomach	
Antrum	2 (7.1)
Angle	2 (7.1)
Lower body	1 (3.6)
Mid body	2 (7.1)
Upper body	1 (3.6)
Duodenum	
Bulb	2 (7.1)
2nd portion	4 (14.3)
Ampulla	6 (21.4)

*ECOG* Eastern Cooperative Oncology Group, *SD* Standard deviation

**Fig. 2:**
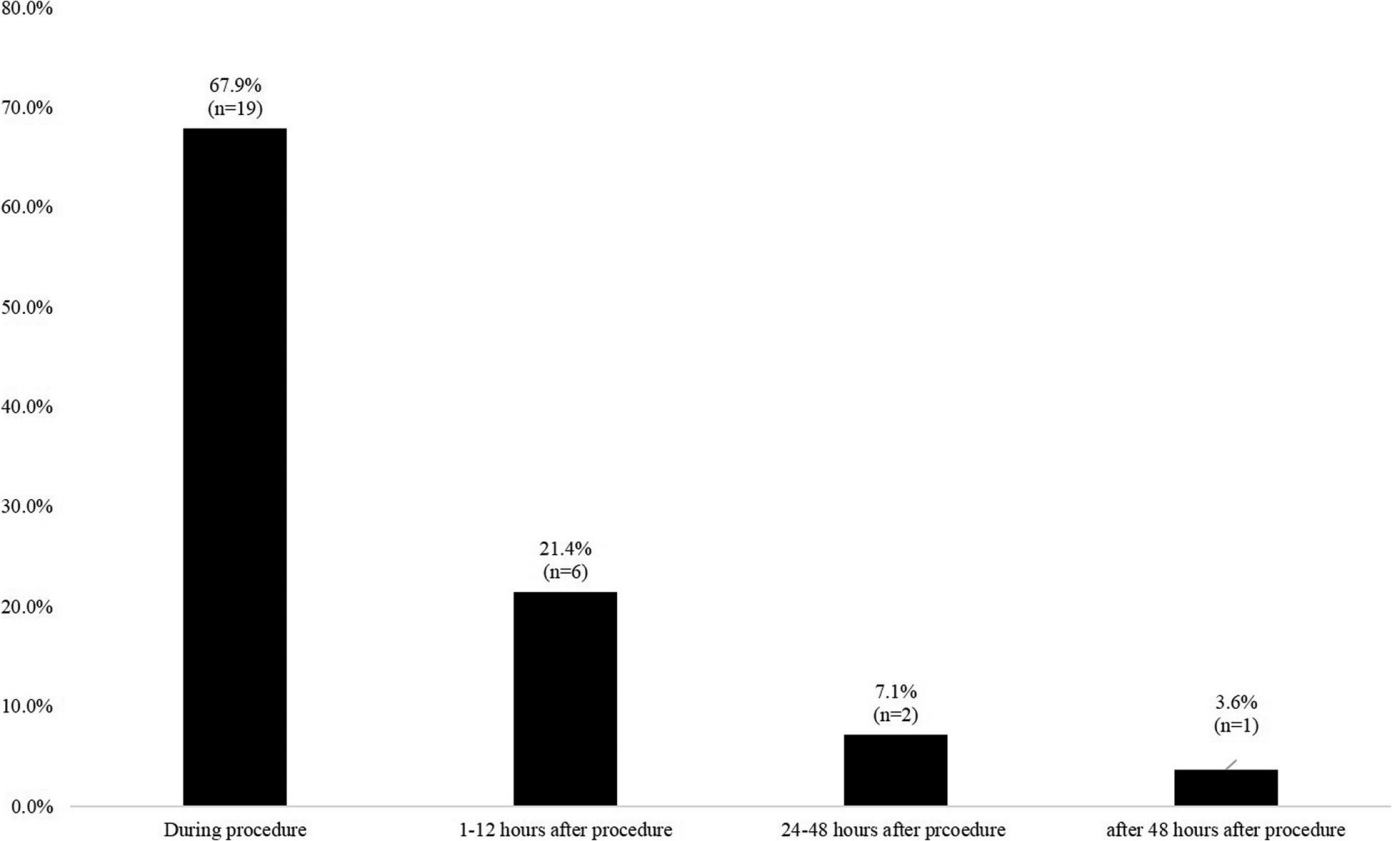
Time of diagnosis of iatrogenic endoscopic perforations

**Fig. 3:**
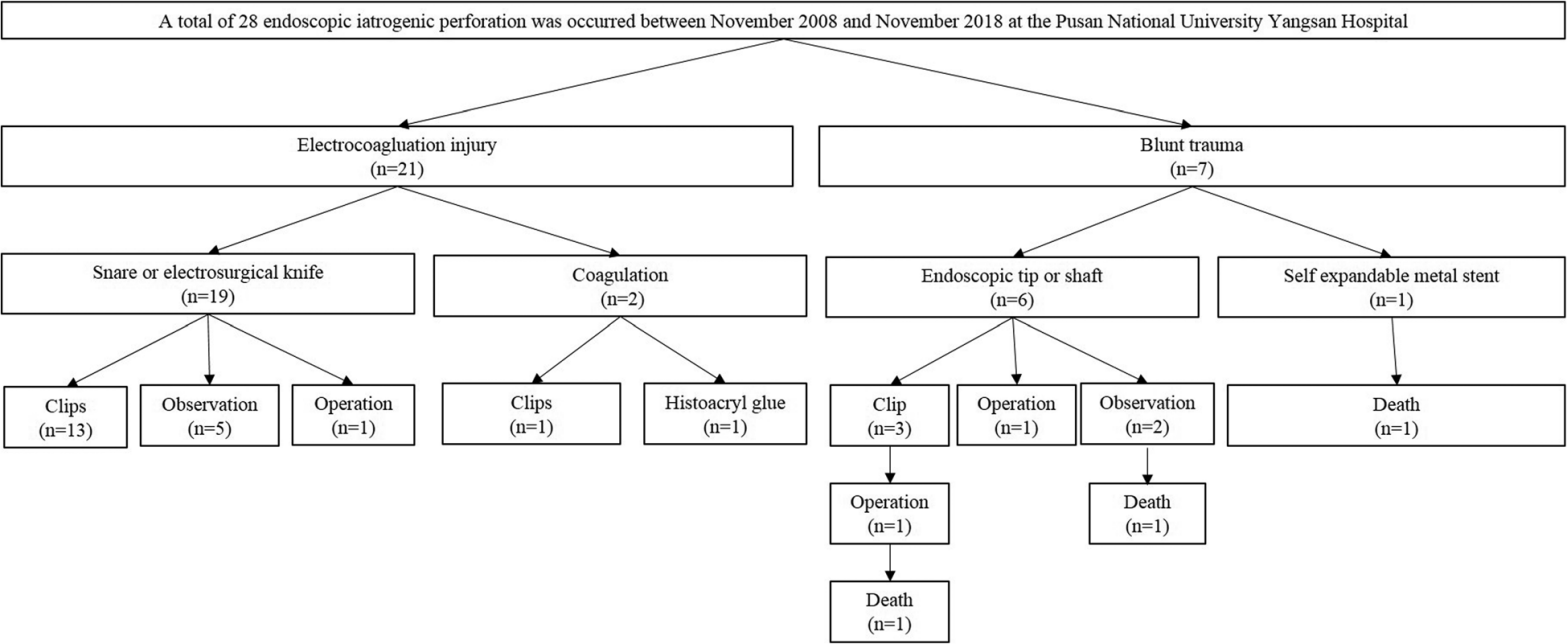
Clinical outcomes based on the mechanisms of iatrogenic endoscopic perforations

With respect to the mechanisms of iatrogenic perforations, electrocoagulation-induced injury (*n* = 21) was more common than blunt trauma-induced injury (*n* = 7). Endoscopic tip- or shaft-induced injuries were common (6/7) among those with blunt trauma-induced perforations, and electrosurgical knife-induced injuries were common among those with electrocoagulation-induced perforations (16/21) (Table 3). Most iatrogenic perforations were diagnosed during endoscopic procedures (67.9%, 19/28) (Fig. 2). Clinical outcomes of iatrogenic endoscopic perforations are shown in Fig. 3. All electrocoagulation-induced perforations were managed successfully (15 cases of endoscopic closure, 5 cases treated conservatively, and 1 case treated surgically), and no mortality was reported. Among patients with blunt trauma-induced iatrogenic endoscopic perforations (*n* = 7), endoscopic closure was attempted in 3 patients using endoscopic clips; however, only 2 perforations were treated successfully. Three patients with advanced malignancy (cholangiocarcinoma, advanced gastric and esophageal cancer) died after blunt trauma-induced perforation. Additional surgical repair was attempted 5 days after the perforation in 1 patient who failed endoscopic closure (ERCP-induced perforation of the 2nd part of the duodenum) because the patient initially refused surgical repair. This patient died 19 days after the iatrogenic perforation. Two patients with advanced malignancy refused surgical repair and died 2–5 days after the iatrogenic perforation (Fig. 3).

**Table 3:** Mechanisms of iatrogenic endoscopic perforations

Type	Number, (%)
Blunt trauma	
Endoscopic tip or shaft-induced injury	6 (21.4)
Self-expandable metal stent-induced injury	1 (3.6)
Electrocoagulation-induced injury	
Snare-induced	3 (10.7)
Electrosurgical knife-induced	16 (57.1)
Coagulation-induced	2 (7.1)

Factors associated with surgical management or mortality were poor performance status (ECOG ≥1), advanced malignancy, a history of gastric surgery, delayed diagnosis, and blunt trauma (Table 4). Factors associated with mortality were old age, poor performance status (ECOG ≥1), advanced malignancy, and blunt trauma (Table 4).

**Table 4:** Factors associated with surgical operation or mortality

	Operation or mortality			Mortality			
	Absence (*n* = 23)	Operation or Mortality (n = 5)	*P* value	Survivors (*n* = 25)	Death (*n* = 3)	Total (*n* = 28)	*P* value
Male, n (%)	12 (82.2)	3 (60.0)	0.75	13 (52.0)	2 (66.7)	15 (53.6)	0.630
Age, mean (SD)	63.8 (17.6)	73.2 (15.4)	0.284	63.2 (17.0)	84.3 (1.52)	65.5 (17.4)	0.045
ECOG			0.004				< 0.001
0	22 (95.7)	2 (40.0)		24 (96.0)	0 (0)	24 (85.7)	
1	0 (0)	1 (20.0)		0 (0)	1 (33.3)	1 (3.6)	
2	1 (4.3)	2 (40.0)		1 (4.0)	2 (66.7)	3 (10.7)	
Advanced cancer, n (%)	1 (4.3)	3 (60.0)	0.001	1 (4.0)	3 (100)	4 (14.3)	< 0.001
History of gastrointestinal operation	0 (0)	2 (40.0)	0.002	1 (4.0)	1 (33.3)	2 (7.1)	0.062
Site of perforation, n(%)			0.146				0.254
Stomach	10 (43.5)	2 (40.0)		11 (44.0)	1 (33.3)	12 (42.9)	
Esophagus	5 (21.7)	3 (60.0)		6 (24.0)	2 (66.7)	8 (28.6)	
Duodenum	8 (34.8)	0 (0)		8 (32.0)	0 (0)	8 (28.6)	
Gross perforation, n(%)	19 (82.6)	3 (60.0)	0.087	19 (76.0)	3 (100)	22 (78.6)	0.632
Delayed diagnosis, n (%)	5 (21.7)	4 (80.0)	0.011	7 (28.0)	2 (66.7)	9 (32.1)	0.175
Blunt trauma, n (%)	2 (8.7)	3 (60.0)	0.007	2 (8.0)	3 (100)	5 (17.9)	< 0.001
Length of hospital stay, (days), mean (SD)	9 (4.3)	14 (10.3)	0.058	10.1 (5.8)	8.6 (9.0)	10.0 (6.0)	0.693

*ECOG* Eastern Cooperative Oncology Group, *SD* Standard deviation

## Discussion

Usually, upper gastrointestinal endoscopic examination is considered safe with a low incidence of iatrogenic endoscopic perforation. However, all endoscopic procedures are necessarily invasive and are associated with a risk of iatrogenic perforation and consequent mortality. Iatrogenic endoscopic perforations may be associated with a long period of hospitalization because the administration of intravenous antibiotics and maintaining a nil per os status is attempted in all patients. Delayed management is associated with iatrogenic endoscopic perforation-related mortality. In recent years, several invasive procedures such as EMR with a conventional snare, ESD with an electrosurgical knife, endoscopic balloon dilation, endoscopic SEMS insertion, and various ERCP-related procedures are commonly used in clinical practice. The National Cancer Screening Program established in Korea ensures that adults aged > 40 years undergo a free biennial endoscopic examination for the evaluation of gastric cancer. In recent times, early detection of tumors is possible owing to the widespread availability of screening endoscopy. In Korea, the detection rate of early gastric cancer was reported to be 57.6% of all gastric cancers [6].

In the present study, iatrogenic esophageal perforations were detected during diagnostic endoscopic examination in 3 patients (0.002%). A previous study reported that the perforation rate during upper gastrointestinal endoscopy was 0.0009–0.01% [2]. In the present study, an esophageal perforation was detected at the cervical esophagus just below the upper esophageal sphincter, and another perforation was detected at the lower esophagus just around the anastomotic site after total gastrectomy. Management using endoscopic clips or SEMS is not technically feasible for perforations at the hypopharyngeal or cervical esophagus, and conservative management with nasogastric tube drainage, withdrawal of oral intake, and parenteral antibiotic administration are the mainstay of treatment. Therefore, early detection of the perforation before contamination of foods or secretion is important. However, in the present study, perforation during endoscopic examination was identified in only 1 patient, and this resolved after conservative management. Another patient developed neck pain and a febrile sensation 3 days after endoscopic examination and showed an abscess at the left neck and needed surgical drainage. In 1 patient with lower esophageal perforation, the perforation was diagnosed a day after the endoscopic examination because the perforation was not identified during the endoscopic procedure. Previous studies have reported that anterior cervical osteophytes, Zenker’s diverticulum, esophageal stricture, malignancies, and duodenal diverticula are factors predisposing to perforation during upper gastrointestinal endoscopy [7, 8]. In the present study, the 3 perforations that occurred during diagnostic endoscopy were all performed by physicians undergoing fellowship training who had been performing endoscopic examination for < 1 year. If the endoscopist encounters resistance when advancing the endoscope from the hypopharynx into the esophagus, withdrawal of the endoscope and detachment from the esophageal wall are warranted to prevent iatrogenic endoscopic perforation.

Although the reported rates of endoscopic balloon dilation-induced perforation were 0.1–0.4% [9], perforations associated with endoscopic balloon dilation performed for benign strictures did not occur in the present study. In another recent study, although non-adherence to “the rule of 3” did not increase the risk of esophageal dilation-induced perforation [10], we tried to adhere to “the rule of 3” to decrease the risk of adverse events associated with esophageal dilation, i.e., the maximum diameter of the dilator (or balloon) used for dilation was not increased by > 3 mm/session [9]. In the present study, perforation associated with endoscopic SEMS performed for malignant esophageal obstruction occurred in 1 patient (0.5%) just after stent placement, and the patient died 2 days post-procedure. Various factors are associated with adverse events after stent placement such as prior administration of chemoradiation, longer SEMS length, and advanced stage tumors [11]. To reduce SEMS placement-related perforation, proper positioning of the guidewire and selection of a stent of appropriate length are important to prevent stent dislocation during stent placement.

The mechanisms associated with iatrogenic endoscopic perforations are electrocoagulation-induced injury during the endoscopic procedure and blunt trauma caused by the endoscope. In recent years, various types of electrosurgical knives and coagulation graspers are used to resect tumors and control gastrointestinal bleeding. Electrocoagulation-induced perforations are smaller in size (< 14 mm) than blunt trauma-induced perforations (approximately 20 mm) [12]. The submucosal or mucosal tissue around the perforated site in electrocoagulation-induced perforations is cleaner and healthier than that around blunt trauma-induced perforations. Therefore, electrocoagulation-induced iatrogenic perforations can be easily repaired endoscopically using clips. In the present study, iatrogenic perforations detected during the endoscopic resection were successfully managed with endoscopic clips or glue in all patients. However, perforation was detected in 1 patient 12 h after an ampullectomy. This patient developed peritonitis and underwent surgical repair. In recent years, ESD is widely used in Korea to treat early gastric cancers and gastric adenomatous lesions. An ESD-associated perforation differs from an EMR-associated perforation in that the former usually occurs as an initial linear tear and undergoes gradual progressive tearing during ESD. Therefore, a perforation observed during ESD warrants prompt closure using clips before further endoscopic resection of the tumor. In contrast, EMR-associated perforations occur as a small round hole after resection of the tumor and can be closed using endoscopic clips [12, 13]. In the present study, all cases of EMR-associated perforations occurred at the duodenum (0.9%). Endoscopic duodenal resection is technically more challenging than endoscopic gastric and esophageal resection because of the following reasons: 1. Owing to the curved and narrow lumen (complicated duodenal anatomy), obtaining and maintaining an adequate visual field is difficult during EMR. 2. Abundant submucosal Brunner’s glands make it difficult to lift the tumor following submucosal injection. Moreover, the muscularis propria layer is thinner than that in the gastric wall. 3. Emergency surgery is warranted in such cases because the tissue damage secondary to bile and pancreatic juice in this segment interferes with adequate wound healing [13, 14]. In the present study, duodenoscope-induced perforations occurred in 0.02% of patients (1 case of esophageal perforation and 2 perforations of the 2nd part of the duodenum). Although 1 esophageal and 1 duodenal perforation could be closed using endoscopic clips, 1 patient failed endoscopic clip closure.

In the present study, the factors associated with surgical management or mortality were poor performance status, advanced malignancy, a history of gastrointestinal operation, delayed diagnosis, and blunt trauma-induced mechanical perforation. Iatrogenic perforations in older patients and in those with a poor performance status and advanced malignancy led to higher mortality rates. This observation could be attributed to the fact that these patients do not tolerate the additional surgical stress. Most patients refused surgical management in the present study because of poor performance status and advanced malignancy.

Limitations of this study are as follows: 1. The retrospective study design is a drawback because a selection bias cannot be excluded. Although the results of this study cannot be generalized, a large number of cases (*n* = 149,792) and a long study period (10 years) may provide useful information for application in clinical settings. 2. Owing to the relatively small number of iatrogenic endoscopic perforations observed in this study, multivariate analysis was not feasible to evaluate the risk factors associated with surgical management or mortality. 3. Over-the-scope clip (OTSC) is useful tool for the treatment of iatrogenic perforation. But we could not use OTSC for closure of the iatrogenic perforations because it is not covered by insurance in Korea during this study period. This may be a limitation that does not reflect the latest trends. 4. And we used carbon dioxide gas in only a few procedures. If carbon dioxide gas was used, the results could be different for perforated patients.

## Conclusion

In summary, diagnostic upper gastrointestinal endoscopy is relatively safe. If resistance is encountered during advancement of the endoscope, particularly at the cervical esophagus, withdrawal of the scope is important along with adequate visualization of the endoscopic field to prevent perforation. Electrocoagulation-induced iatrogenic perforations can be closed with endoscopic clips in most cases. However, if blunt trauma occurs secondary to the endoscope tip or shaft, prompt endoscopic closure of such perforations is difficult, and prompt surgical management is recommended in patients in whom endoscopic closure fails. Patients with a microperforation without evidence of infection or sepsis can undergo conservative management with the administration of parenteral antibiotics and nutritional support.

## Data Availability

The datasets used and/or analysed during the current study are available from the corresponding author on reasonable request.

## Abbreviations

ECOG: Eastern Cooperative Oncology Group
EMR: Endoscopic mucosal resection
ERCP: Endoscopic retrograde cholangiopancreatography
ESD: Endoscopic submucosal dissection
SEMS: Self-expandable metal stent
